# Stressing the Relevance of Differentiating between Systematic and Random Measurement Errors in Ultrasound Muscle Thickness Diagnostics

**DOI:** 10.1186/s40798-024-00755-z

**Published:** 2024-08-15

**Authors:** Lars Hubertus Lohmann, Martin Hillebrecht, Stephan Schiemann, Konstantin Warneke

**Affiliations:** 1 University Sport Center, Carl von Ossietzky University Oldenburg, Oldenburg, Germany; 2 Department of Human Movement Science and Exercise Physiology, Institute of Sport Science, Friedrich Schiller University, Jena, Germany; 3 Institute of Exercise, Sport and Health, Leuphana University, Lüneburg, Germany; 4 Institute of Sport Sciences, University of Klagenfurt, Klagenfurt am Wörthersee, Austria

**Keywords:** Ultrasound, Reliability, Training intervention studies, Mean absolute percentage error, Expected effect

## Abstract

**Background:**

The majority of studies that explore changes in musculature following resistance training interventions or examine atrophy due to immobilization or sarcopenia use ultrasound imaging. While most studies assume acceptable to excellent reliability, there seems to be unawareness of the existing absolute measurement errors. As early as 1998, methodological research addressed a collective unawareness of the random measurement error and its practical indications. Referring to available methodological approaches, within this work, we point out the limited value of focusing on relative, correlation-based reliability indices for the interpretability in scientific research but also for clinical application by assessing 1,512 muscle thickness values from more than 400 ultrasound images. To account for intra- and inter-day repeatability, data were collected on two consecutive days within four testing sessions. Commonly-stated reliability values (ICC, CV, SEM and MDC) were calculated, while evidence-based agreement analyses were applied to provide the accompanied systematic and random measurement error.

**Results:**

While ICCs in the range of 0.832 to 0.998 are in accordance with the available literature, the mean absolute percentage error ranges from 1.34 to 20.38% and the mean systematic bias from 0.78 to 4.01 mm (all *p* ≤ 0.013), depending on the measurement time points chosen for data processing.

**Conclusions:**

In accordance with prior literature, a more cautious interpretation of relative reliability values should be based on included systematic and random absolute measurement scattering. Lastly, this paper discusses the rationale for including different measurement error statistics when determining the validity of pre-post changes, thus, accounting for the certainty of evidence.

## Key Points

• While reliability of a testing protocol is most often determined via relative reliability indices such as the intraclass correlation coefficient, further reliability values such as the systematic bias and the random error have been described as valuable for results interpretation.

• This study used the most frequently employed procedure to determine muscle hypertrophy (ultrasound) as an example linking relative reliability values to absolute measurement errors under special consideration of appropriate calculation models using three scenarios (best case, worst case, stability).

• Overall, 504 ultrasound images were examined showing excellent relative reliability, but the corresponding measurement errors suggest that caution must be exercised when interpreting pre-post settings in cases where the measurement error exceeds the expected changes.

## Background

Due to its high importance in rehabilitation and prevention, several exercise training programs were designed to induce muscle hypertrophy in healthy participants or after injury [1], while the muscle thickness/cross-sectional area are considered of utmost importance when quantifying age-related sarcopenia [2].

Assuming training-induced muscle mass increases of 7.6 ± 1.2% (d = 0.47 ± 0.08) in intervention periods of up to 13 weeks [3], a highly sensitive, and therefore reliable as well as reproducible procedure for data collection is strongly recommended in sports medicine and science to preclude measured differences being the result of measurement errors [4]. While described as the gold standard method, magnetic resonance imaging [5] is frequently substituted by ultrasound muscle thickness evaluations as the literature suggests high validity and reliability, while being portable and cost- as well as time-efficient [5–7].

Notwithstanding, concerns arose regarding the objectivity of using ultrasound due to applied pressure to soft tissue, lack of probe angle standardization and lack of agreement with muscle cross-sectional area values from magnetic resonance imaging [8]. As early as 1998, Atkinson & Nevill [9] as well as Lamb [10] drew attention to unsatisfactory reliability when validating measurement procedures. Additionally, de Vet et al. [11] as well as Kottner et al. [12] highlighted that the context of a given measurement set-up is of utmost importance, stressing the relevance of using agreement and not reliability measures to quantify the magnitude of measurement error when evaluating changes over time.

Even though 25 years have passed since Atkinson & Nevill [9] as well as Lamb [10] published their respective papers, it appears that an unawareness of the detailed quantification and evaluation of systematic and random measurement errors still exists in sports medicine and science. This is because reliability and repeatability are most often solely stated on the basis of correlations (i.e. intraclass correlation coefficient (ICC)) and its derivatives (such as the standard error of the mean (SEM) or minimal detectable change (MDC)) [4, 13] as can be seen in a systematic review concerned with ultrasound reliability by Nijholt et al. [7]. Relative reliability indices, expressed as correlation coefficient-based statistical parameters, focus on the relationship between two values with or without accounting for variance and do not distinguish (in a sense of separate quantification) between systematic and random error [4, 9].

For all users of a specific measurement method, practitioners such as therapists and medical staff or researchers, it is of paramount importance to be able to distinguish systematic bias (error arising from, e.g., habituation, familiarization or in ultrasound from muscle swelling or water content increases) from random error (unsystematic scattering from, for example, different probe pressure or angle) when interpreting results [4, 13]. While commonly-used reliability indices seem relevant for assessing relative reliability [4], Lamb [10] has impressively delineated the limitations of correlation-based reliability calculation methods for interpretability regarding the repeatability of the testing procedure.

Therefore, in this study, we aimed to apply the commonly used (also considered standard) methods for reliability calculations in sports science and medicine research and oppose these methods to those proposed, inter alia, in the articles by Barnhart et al. [4], Hopkins [13] and Atkinson & Nevill [9]. Therefore, after firstly calculating the ICC, SEM and MDC with those formulas most commonly employed in current original sports science and medicine research, secondly, the corresponding systematic and random errors, arising from test-retest performance (i.e., intra- and inter-day reliability), will be provided to raise awareness of the strengths strengths and weaknesses of the commonly used reliability reporting methods.

Accordingly, to provide a well-balanced perspective on the repeatability and stability of ultrasound muscle thickness data collection, three different scenarios of measurement error calculation will be presented, stressing the relevance of reporting the random error when performing diagnostics.

## Methods

### Experimental Set-up

Data collection was performed on two consecutive days, including 2 test sessions each day (4 test sessions in total), while assuming no meaningful exercise-induced morphological adaptations within 48 h. Muscle thickness images were acquired via B-mode ultrasound once in the morning and once in the (late) afternoon of both these days. The muscles investigated are the vastus lateralis (VL), the lateral head of the gastrocnemius (GL) and the medial head of the gastrocnemius (GM) – chosen as these exhibit some of the highest ICC values stated in the literature for ultrasound muscle thickness measurements and are frequently investigated in training intervention studies [6, 14, 15].

**Fig. 1:**
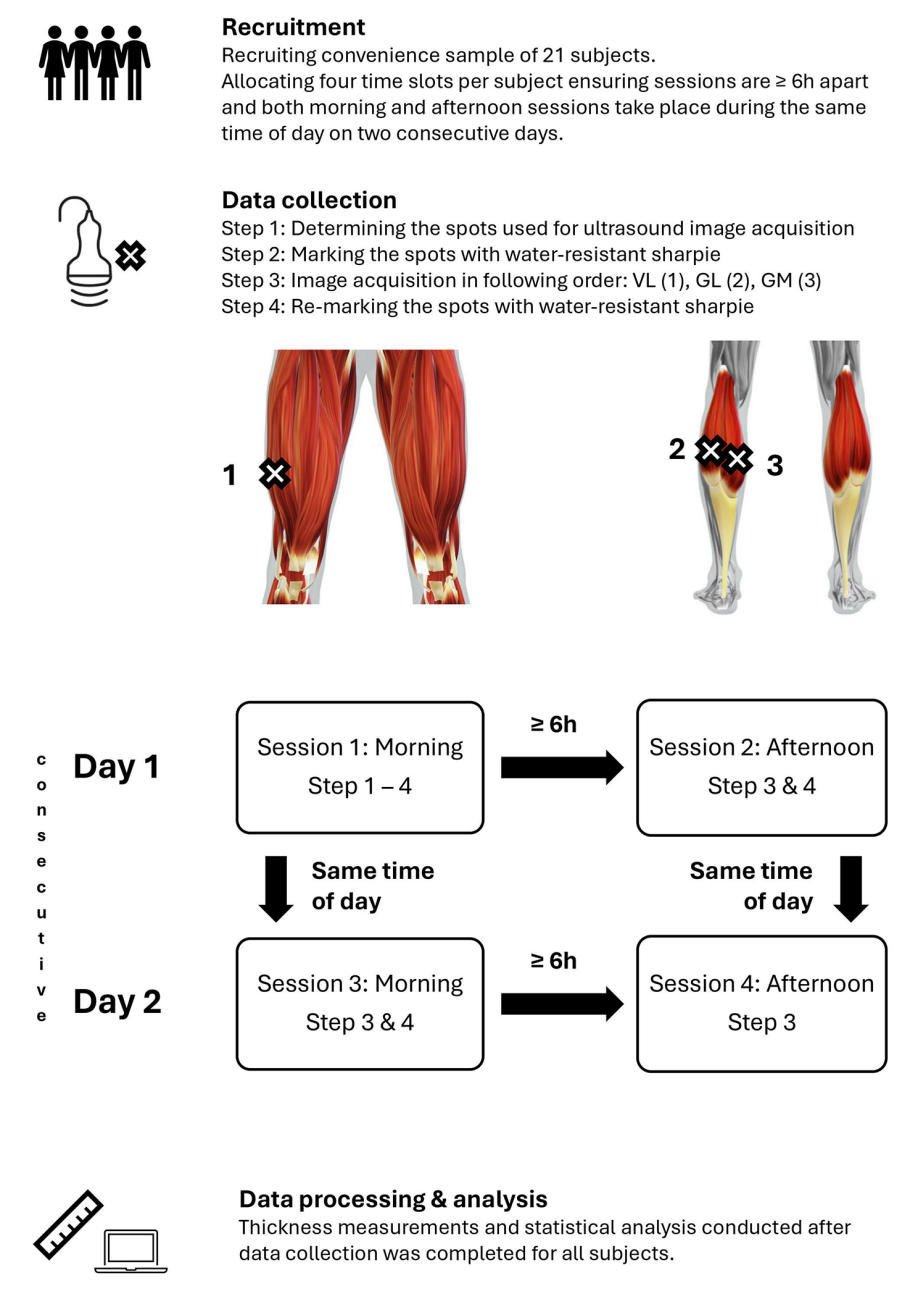
Flow-chart showing how the experiment was conducted

In total, 504 images from 21 participants (see *Image acquisition* and *Participants* sections) and thus 168 images per muscle were used for the calculation (21 participants × 4 measurement time points × 3 muscles × 2 images per muscle). Since all images comprised three muscle thickness determinations across the width of the image (left, middle, right), the calculations are based on a total of 1,512 muscle thickness determinations. Data were collected by the same experienced investigator (LHL) who has been involved in extensive B-mode ultrasound image acquisition for muscle thickness determination in various chronic static stretching intervention studies [16, 17]. Figure 1 shows a flow-chart illustrating how the experiment was conducted.

### Image Acquisition

The B-mode ultrasound images were acquired using a MyLab™ Gamma ultrasound device with a 5 cm wide SL1543 linear probe (Esaote Biomedica DE GmbH, Cologne, Germany) operating at a frequency range of 3 to 13 MHz with image acquisition in the longitudinal direction. To ensure using the same spots for the repeated measurements, all spots were marked with a water-resistant sharpie and re-painted in each session.

For VL measurements (in the right leg), the participants adopted a seated position with the knees slightly over the edge of a massage bench to ensure no contraction in the quadriceps musculature. For GM and GL measurements (in the left leg), the participants assumed a prone position on the same massage bench with their feet hanging slightly over the edge of the bench to ensure no contraction in the calf muscles.

Muscle thickness was defined as the distance between the superficial and deep aponeuroses of a muscle. The spots used for the ultrasound muscle thickness measurements on the right VL as well as left GL and GM were determined following two criteria: (1) clear image and (2) superficial and deep aponeuroses as parallel as possible to ensure that the measurement point was not close to a muscle-tendon junction.

**Fig. 2:**
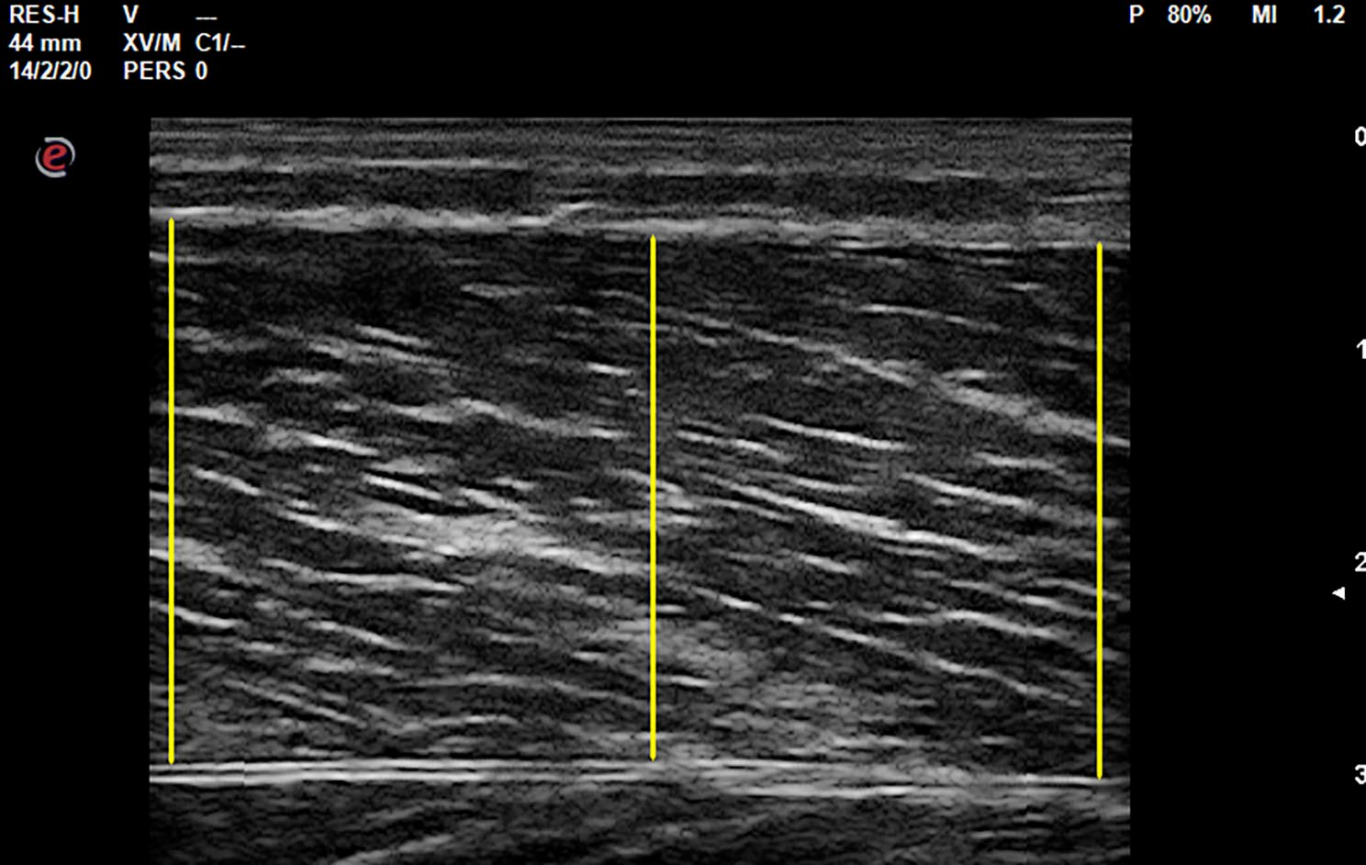
Illustration of how three distances between the upper and lower fascia of the respective muscles across the width of an image (left, middle, right) were used to determine the mean muscle thickness for each acquired image

To counteract potential variations within a single ultrasound picture and minimize assessment limitations, for each muscle, muscle thickness was calculated as the mean of three distances between the upper and lower fascia in each picture, leading to 1,512 muscle thickness values (504 pictures x 3 muscle thickness determinations). Image processing was performed via ImageJ (version 1.53t, National Institutes of Health, Bethesda, MD, USA) which is illustrated in Fig. 2.

### Participants

To account for the widespread application in different clinical settings including heterogeneous performance level, sexes/gender and anthropometric parameters, the included participants’ attributes ranged from sedentary lifestyle with no training history to strength training seven days per week (bodybuilder with a body mass of 125 kg). Therefore, the age, height, mass and body mass index ranged from 20 to 65 years (33.9 ± 14.2 years), 168 to 195 cm (180.71 ± 7.34 cm), 66 to 130 kg (86.95 ± 19.5 kg) and 21.46 to 40.12 kg/m^2^ (26.48 ± 4.9 kg/m^2^), respectively, for the 13 male and 8 female participants. All participants provided written informed consent for participation in the study which was conducted in accordance with the Declaration of Helsinki and approved by the Oldenburg Medical Ethics Committee (2021-089).

### Data Analysis

In the first step, commonly-used reliability parameters were calculated using SPSS 29 (IBM Deutschland GmbH, Ehningen, Germany) and Microsoft Excel (Microsoft Corp., Redmond, WA, USA). These include:

1. ICC two-way mixed model for consistency [18].

1 $$\text{ICC}=({\text{MS}}_{\text{R}}-{\text{MS}}_{\text{E}})/{\text{MS}}_{\text{R}}$$

2) ICC two-way mixed effects model for absolute agreement [18].

2 $$\text{ICC}=({\text{MS}}_{\text{R}}-{\text{MS}}_{\text{E}})/\left({\text{MS}}_{\text{R}}+\left({\text{MS}}_{\text{C}}-{\text{MS}}_{\text{E}}\right)/\text{n}\right)$$

3) The coefficient of variability (CV).

3 $$CV=\frac{SD}{Mean}*\:100$$

4) The standard error of measurement for consistency (SEM_consistency_).

4 $${SEM}_{consistency}=SD*\sqrt{1-{\text{ICC}}_{\text{consistency}}}$$

5) The SEM for agreement (SEM_agreement_).

5 $${\text{SEM}}_{agreement}=\sqrt{{({\sigma}}_{observations}^{2}+{{\sigma}}_{residual}^{2})}$$

6) The minimal detectable change (MDC).

6 $$\text{MDC}=\text{SEM}*1.96*\sqrt{2}$$

The terms used in the above equations are: ICC = intraclass correlation coefficient, ${\text{MS}}_{\text{C}}$ = mean square for columns, ${\text{M}\text{S}}_{\text{E}}$ = mean square for error, ${\text{MS}}_{\text{R}}$ = mean square for rows, n = number of subjects, SD = standard deviation, ${{\sigma}}_{observations}^{2}$ = the variance in observations, ${{\sigma}}_{residual}^{2}$ = residual variance being the interaction between subjects and observations

Noteworthy, the SEM and MDC share most of the measures with the ICC as they are all based on variability values that stem from analysis of variance (ANOVA) calculations. While the SEM_consistency_ and the “consistency”-based MDC are not suitable to assess agreement between measurements, they are still commonly used within sports science and medicine research (see, e.g., [19–22]). Therefore, these parameters are included in the following analyses and supplemented by the “agreement”-based variations. The MDC is generally calculated with the same formula irrespective of its use being within consistency or agreement settings and will be listed separately.

In advance of the following calculations, the construct of reliability must be discussed. We aimed to explore repeatability as the basis of all further reliability models, meaning that the same investigator assessed the same parameter on the same subject, just at a different time point. Assuming no further variation in the testing conditions, using a reliable and valid measurement tool, maximal *agreement* between the values can reasonably be assumed. Accordingly, Barnhart, Haber & Lin [4] provide an overview of different assumptions and calculation models to assess repeatability in measurements. To account for the random error, including the variance of individual courses providing a range of the typical error, Hopkins [13] described it as the mean of the standard deviations (SD) divided by $\sqrt{2}$. Assuming heteroscedasticity in most sports science and medicine-related parameters, the absolute typical error (TE) usually increases with higher performance levels [23], and the statement of the percentage of the TE can thus be assumed beneficial [13]. Therefore, the TE as well as the CV of the TE (CV_TE_) are also provided in Table 1. A further agreement analysis considering the individual deviations of the mean was provided by Bland & Altman [24], graphically illustrating the systematic bias (which is equal to the mean differences of the paired t-test applied to the data of interest) with the scatter of individual plots. Furthermore, the limits of agreement (LoA) are included to this graphical evaluation. Consequently, to assign the TE, mean absolute error (MAE), the mean absolute percentage error (MAPE) as well as the graphical illustration of the random error to the commonly stated reliability measures, these values were additionally added to Table 1. The level of significance for the mean systematic bias via paired t-test was set at *p* < 0.05.

**Table 1:** Absolute error statistics based on the ultrasound-derived muscle thickness values acquired during four measurement time points on two consecutive days

Muscle	Comparison	CV (in %)	TE (in mm)	CV_TE_ (in %)	MAE (in mm)	MAPE (in %)	Mean systematic bias (95% CI) (in mm)	LoA of mean systematic bias (95% CI) (in mm)
**VL**	Highest vs. sec. highest	1.96	0.92	3.45	0.77	2.67	0.78 (0.18, 1.37) *p* = 0.013*	-1.78 (-2.81 – -0.75) – 3.33 (2.3–4.36)
	Highest vs. lowest	11.79	1.35	5.06	4.06	15.11	4.01 (3.19, 4.93) *p* < 0.001**	0.32 (-1.19–1.83) – 7.8 (6.29–9.31)
	Highest vs. mean	5.61	0.923	3.46	2.06	7.54	2.06 (0.28, 1.47) *p* < 0.001**	-0.5 (-1.53–0.53) – 4.62 (3.59–5.65)
**GL**	Highest vs. sec. highest	3.36	0.358	2.37	0.69	4.58	0.69 (0.46, 0.92) *p* < 0.001**	-0.3 (-0.7–0.1) – 1.68 (1.28–2.08)
	Highest vs. lowest	16.27	0.729	4.83	3.23	20.38	3.24 (2.77, 3.7) *p* < 0.001**	1.21 (0.4–2.03) – 5.26 (4.44–6.07)
	Highest vs. mean	8.2	0.459	3.04	1.71	10.86	1.71 (1.41, 2) *p* < 0.001**	0.43 (-0.08–0.95) – 2.98 (2.47–3.5)
**GM**	Highest vs. sec. highest	0.96	0.239	1.14	0.31	1.34	0.31 (0.15, 0.46) *p* < 0.001**	-0.36 (-0.62 – -0.09) – 0.97 (0.7–1.24)
	Highest vs. lowest	6.25	0.942	4.51	1.91	8.39	1.91 (1.3, 2.51) *p* < 0.001**	-0.71 (-1.76–0.35) – 4.52 (3.46–5.57)
	Highest vs. mean	3.04	0.544	2.6	0.97	4.18	0.97 (0.62, 1.32) *p* < 0.001**	-0.54 (-1.15–0.07) – 2.48 (1.87–3.09)

ICC = Intraclass correlation coefficient, CV = Coefficient of variation, TE = Typical error, CV_TE_ = Typical error expressed as a coefficient of variation, MAE = Mean absolute error, MAPE = Mean absolute percentage error, LoA = Limits of agreement, (95% CI) = 95% confidence interval, Highest = Highest muscle thickness value, sec. highest = Second highest muscle thickness value, lowest = Lowest muscle thickness value, mean = Mean muscle thickness value across eight images, * = significant *p* < 0.05, ** = significant *p* ≤ 0.001

7 $$\text{MAE}=\frac{1}{\text{n}}*\sum\nolimits_{\text{i}=1}^{\text{n}}\left|{\text{x}}_{\text{i}}-{\text{y}}_{\text{i}}\right|$$

8 $$\text{MAPE}=\frac{1}{n}*\sum\nolimits_{i=1}^{n}\left|\frac{{x}_{i}-{y}_{i}}{{x}_{i}}\right|*100$$

9 $$\text{TE}=\text{SD}({\text{x}}_{\text{i}}-{\text{y}}_{\text{i}})/\sqrt{2}$$

10 $${\text{CV}}_{\text{TE}}=\frac{\text{TE}}{\text{Mean}}*100$$

The terms used in the above equation are: n = number of subjects, $\:{\text{x}}_{\text{i}}=\text{value}\:\text{of}\:\text{measurement}\:1$, ${\text{y}}_{\text{i}}=\text{value}\:\text{of}\:\text{measurement}\:2$, SD = standard deviation, TE = typical error

The Bland-Altman plot stems from the JAMOVI software (version 2.3.28) using the ‘blandr’ module.

To control the data for a possible influence of bodyfat on imaging quality, the Pearson product-moment correlation coefficient values for the subgroups *normal body-mass-index* vs. *overweight* as well as *male* vs. *female* were z-transformed according to the Fisher method. The Benjamini-Hochberg procedure was used to control the study-wise false discovery rate with a significance value of 0.05 [25]. The analysis yielded no significant differences in relationships of these parameters for the subgroups. Additionally, the review by Nijholt et al. [7] found no differences in the reliability of ultrasound measurements between older and younger participants.

### Three Scenario Calculation

Commonly, reliability values are calculated using the best and the second-best value available (scenario 1). However, when aiming to provide objectively reported results for practical useful information [26], this procedure can be exclusively performed in very stable measurement procedures, especially if the real muscle thickness is not known and can exclusively be determined by using the performed procedure [4]. To illustrate, when measuring a muscle thickness of 5 mm in trial 1 and 6 mm in trial 2, can we assume the real value to be 5 mm, 6 mm (20% increase compared to trial 1) or 5.5 mm? Since the real values are unknown, the stability of the measurement provides a range of the true measurement errors, leading to a statement about the precision of the measurement. However, from a scientific point of view, we cannot exclusively use the best-case scenario but should also consider the probability of personal errors. Thus, a well-balanced perspective requires providing the worst-case scenario as well (scenario 2). Additionally, accounting for the stability of the measurement (and to weaken the worst-case scenario), our third scenario provides the best measurement value compared to the mean across all measurement values (scenario 3). The best and worst measurements represent the highest and lowest muscle thickness values, respectively.

## Results

Table 2 reports the descriptive statistics for the muscle thickness measurements.

**Table 2:** Muscle thickness measurement characteristics

Muscle	*N*	Total number of images	Min (in mm)	Max (in mm)	M (95% CI) ± SD (in mm)
Vastus lateralis	21	168	11.7	53.4	26.66 (25.25, 28.07) ± 9.26
Lateral head of gastrocnemius	21	168	6.8	30.1	15.1 (14.26, 15.93) ± 5.55
Medial head of gastrocnemius	21	168	14.3	37.2	20.89 (20.14, 21.64) ± 4.91

N = Number of participants, Min = Minimal muscle thickness value, Max = Maximal muscle thickness value, mm = Millimeter, M ± SD = Mean ± standard deviation

### Best-case Scenario (Scenario 1)

Using the best and second-best value, the best-case scenario exhibits ICCs for agreement and consistency that would be classified as excellent according to the current literature, ranging from 0.988 to 0.998 with CV values from 0.96 to 3.36% and SEMs ranging from 0.01 to 0.12 mm for consistency and from 1.03 to 2.67 mm for agreement measures. Since the MDC is strongly related to the SEM, just multiplied by a fixed factor, we do not additionally list this value but report it in Table 3 only. The listed relative reliability values correspond, exemplarily for the VL, to an absolute, systematic measurement error (mean systematic bias) based on the test-retest procedure of 0.78 mm (*p* = 0.013) with LoAs ranging from − 1.78 to 3.33 mm. Therefore, accounting for the random individual scattering, the typical error occurring from repeated measurements calculated in the best-case scenario for VL, GL and GM shows noise of 0.239–0.92 mm for TE, 0.31–0.77 mm for MAE and 1.34–4.58% for MAPE. Test-retest values with LoAs for GL and GM as well as 95% confidence intervals are reported in Tables 1 and 3.

**Table 3:** Relative, correlation-based reliability based on the ultrasound-derived muscle thickness values acquired during four measurement time points on two consecutive days

Muscle	Comparison	ICC_consistency_ (95% CI)	ICC_agreement_ (95% CI)	SEM_consistency_ (in mm)	SEM_agreement_ (in mm)	MDC_consistency_ (in mm)	MDC_agreement_ (in mm)
VL	Highest vs. sec. highest	0.991 (0.978, 0.996)	0.988 (0.96, 0.996)	0.12	2.67	0.34	7.41
	Highest vs. lowest	0.98 (0.951, 0.992)	0.898 (0, 0.978)	0.27	13.23	0.75	36.66
	Highest vs. mean	0.991 (0.977, 0.996)	0.968 (0.347, 0.993)	0.12	6.74	0.34	18.68
GL	Highest vs. sec. highest	0.996 (0.99, 0.998)	0.988 (0.75, 0.997)	0.03	2.26	0.09	6.27
	Highest vs. lowest	0.982 (0.955, 0.993)	0.832 (0, 0.964)	0.14	10.51	0.38	29.13
	Highest vs. mean	0.993 (0.983, 0.997)	0.947 (0.019, 0.99)	0.05	5.55	0.15	15.39
GM	Highest vs. sec. highest	0.998 (0.995, 0.999)	0.997 (0.974, 0.999)	0.01	1.03	0.04	2.84
	Highest vs. lowest	0.965 (0.916, 0.986)	0.903 (0.132, 0.976)	0.25	6.25	0.69	17.32
	Highest vs. mean	0.989 (0.973, 0.996)	0.973 (0.609, 0.993)	0.08	3.19	0.22	8.84

ICC = Intraclass correlation coefficient, SEM = Standard error of measurement, MDC = Minimal detectable change, (95% CI) = 95% confidence interval, Highest = Highest muscle thickness value, sec. highest = Second highest muscle thickness value, lowest = Lowest muscle thickness value, mean = Mean muscle thickness value across eight images

### Worst-case Scenario (Scenario 2)

In contrast to the best-case scenario, scenario 2 compare the highest and lowest muscle thickness values, which, logically, exhibits the largest deviation in muscle thickness measured within the two days and four measurement time points. These comparisons still result in relative reliability with ICCs ranging from 0.965 to 0.982 for consistency and 0.832 to 0.903 for agreement with CV values from 6.25 to 16.27% and SEMs ranging from 0.14 to 0.27 mm for consistency and from 6.25 to 13.23 mm for agreement. The systematic bias calculated via test-retest procedure and Bland-Altman analysis yields a 3.24 mm (*p* < 0.001) difference with LoA of 2.77–3.7 mm for the GL. The calculated TE shows an expected range of 0.729–1.35 mm, while MAE and MAPE are quantified in ranges of 1.91–4.06 mm and 8.39–20.38%, respectively. Test-retest values with LoA for GL and GM as well as 95% confidence intervals are reported in Tables 1 and 3.

### Measurement Stability (Scenario 3)

Hypothesizing both previous scenarios to be unrealistic and in an attempt to include measurement stability, the third scenario uses the best value and the mean of the measurements, resulting in ICCs ranging from 0.989 to 0.993 for consistency and 0.947 to 0.973 for agreement with CV values from 3.04 to 8.2% and SEMs ranging from 0.05 to 0.12 mm for consistency and from 3.19 to 6.74 mm for agreement. The test-retest procedure and Bland-Altman analysis states a systematic measurement bias of 2.06 mm (*p* < 0.001) with LoA of 0.28–1.47 mm. For VL, GL and GM, the TEs range from 0.239 to 1.35 mm, MAEs from 0.97 to 2.06 mm and MAPEs from 4.18 to 10.86%. Test-retest values with LoA for GL and GM as well as 95% confidence intervals are reported in Tables 1 and 3.

**Fig. 3:**
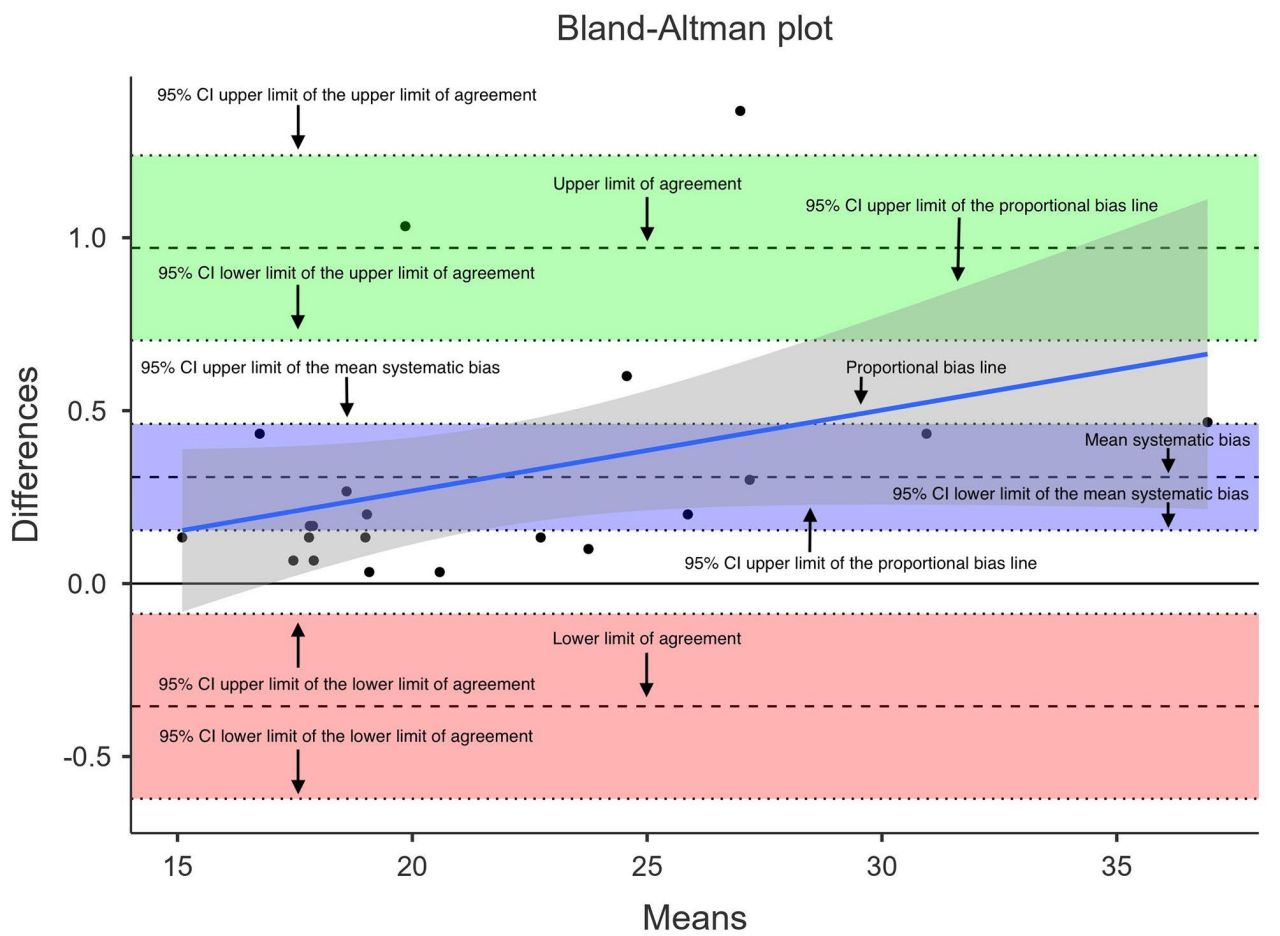
Bland-Altman plot for the best-case scenario of the medial head of the gastrocnemius. The quantification of the systematic error (mean difference) as well as the random scattering that illustrates the random error/secondary variance provide crucial information beyond information on relative reliability. In accordance with Carstensen et al. [27], the limits of agreement provide a range in which 95% of the measurements could be expected when repeating the measurement via the same devices in the same population. Especially the random error should be considered as highly important in ultrasound as it might indicate unsystematic standardization problems (e.g., different probe angles, different measurement spots, differences in applied pressure [8]), while the systematic error could be attributed to, for example, muscle swelling or increased water content in measurements conducted in the evening. Systematic bias = mean difference between mean 1 and mean 2, random error = scattering around the systematic error, lower and upper limit of agreement = reference interval or normal range for the test-retest differences expected for 95% of individuals causing a probability statement for expected values [28]

Figure 3 provides an example for the best-case scenario for GM as a Bland-Altman plot.

## Discussion

Around 20 to 25 years ago, several authors [4, 10] had already stressed the paramount importance of not focusing solely on relative errors, considering means and standard deviations, but rather shifting the focus to random measurement errors, especially when addressing clinical and practical applicability. However, the majority of the literature still almost exclusively reports ICCs (sometimes the CV), the SEM/MDC, while collectively neglecting the random scattering of individual value pairs, arising from repeated measurements. Consequently, the present study was designed to evaluate the commonly-used relative error values and to additionally provide recommended random error parameters. With ICCs ranging from 0.832 to 0.998, the data collection showed comparable reliability to the current ultrasound literature [7]. Depending on the scenario, we found significant (all *p* < 0.05 and all but one *p* < 0.001) systematic error as represented by the mean systematic bias and the corresponding LoA despite the small sample size.

In scientific settings as well as in clinical practice, the use of precise and accurate measurements is of critical importance. The criteria objectivity, validity and reliability are commonly known as preconditions for the further use of collected data. However, there seems to be no consensus about the classification of the aforementioned criteria. While mostly referring to Cohen’s [29] classifications, it seems that authors neglect important aspects. Firstly, the suggested classifications are based on assumptions from mostly behavioral and psychological sciences. Secondly, it is clearly described that classifications of reliability must always be viewed in the light of the setting in which they are applied [30, 31]. Using correlation-based reliability values, it seems reasonable to consider two aspects. On the one hand, as mentioned above, the true value is not known. Consequently, the better the reliability, the closer the LoA of the measurements and, thus, the scatter range of individual deviations decreases. On the other hand, the expected or measured pre-post change of a measurement tool provides the relevance of the random measurement error, as the systematic measurement error (a mean error shift over- or under-calculating the real value by repeating the measurement without surrounding scattering) could be solved by adding a fixed factor to the formula. Therefore, relating to reliability, repeatability (intra- and inter-day reliability) can be described as a value of measurement precision and vice versa measurement precision a value of repeatability [4]. Additionally, as already described by Lamb [10], using a measurement tool with a systematic and random error can not be assumed to be either objective or valid. Nevertheless, it is still mandatory to attribute measured noise as well as systematic bias to the related circumstances and context.

Random errors in ultrasound include differences in water content in the musculature due to variations in hydration, activity level on the measurement day and possibly the days prior but also the applied pressure with which the transducer is placed on the skin, sub-optimal standardization of the measurement point etc [8]. This list is not exhaustive but already highlights many different possible influences affecting the outcome.

Regardless of the resulting measurement error, the further relevance of the variability magnitude in sports science arises from the expected increase in intervention studies. The literature indicates muscle hypertrophy effects at around 7.6 ± 1.2% in response to up to 13 weeks of resistance training [3] while Goodpaster et al. [32] quantified the age-related loss of skeletal muscle mass to be around 1% per year within a 3-year span in a study sample of 1,880 subjects with a mean age of 73.5 ± 2.8 years. Even though most intervention studies are controlled via a passive control group and assuming no statistically significant changes from pre- to post-test (in which the same measurement error could be assumed), the repeatability values might not be sufficient to prove a difference between groups in general, implicating that a more cautious interpretation of increases is needed. Therefore, contrasting the measurement errors of the best-case, worst-case and stability scenario to changes of 7.6 ± 1.2% in resistance-training studies and 1% per year in sarcopenia-related atrophy, the question arises about the real pre-post changes.

When drawing conclusions on a bigger scale, this would encourage rating reliability on agreement measures (such as absolute agreement ICCs) and adjusting the classification based on the expected effect (size) as well as the expected measurement error, which would make the assessment more meaningful. A similar approach is already in effect in meta-analyses and other review articles when quality of evidence and strength of recommendation are judged based on a framework. A good example is the renowned GRADE framework [33] that first grades the level of evidence as high for randomized trials, low for observational studies and very low for any other evidence, after which the level is adjusted, decreasing, e.g., with serious limitations in study quality, imprecise data or high probability of reporting bias, but also increasing inter alia with strong evidence of association or evidence of a dose-response gradient.

Grading the ICC values based on measures of error makes it a necessity to consider the setting in which the measurement takes place. A 7.54% MAPE should be considered too high when assessing muscle thickness/cross-sectional area via ultrasound for pre-post-comparisons in short-lasting training interventions but could be negligible, e.g., when measuring the maximal strength in the squat in a one-year strength training study in previously untrained subjects where much higher effects are to be expected. Potentially, this could contribute to researchers critically questioning and appraising reliability classifications and their own work instead of unreflectively following the current conventions. Additionally, in turn, whether a measurement error is high or low might be relativized by the magnitude of the effect. Therefore, the LoAs in Bland-Altman analyses should be defined prior to an investigation when determining a tolerable range. When referring back to their original application to evaluate the agreement between blood pressure devices, Bland & Altman [24] performed exactly this procedure. In regard to reliability, Wright & Royston [28] defined the LoAs as the reference interval for test-retest differences expected for 95% of individuals. Thus, it can be considered the range most of the measurement errors will fall into when repeating the testing procedure under equal conditions in the same population [27]. Consequently, the evaluated LoA span can be used to check if testing was performed under suitable conditions meaning the error did not surpass the pre-defined ranges. Currently, it seems that these parameters are regularly determined without any consequence for the interpretation of the following results.

### Limitations

This study’s operator (LHL) acquired and rated all ultrasound images with utmost care. However, it cannot be precluded that investigator-dependent errors occurred, which might be present in any ultrasound investigation. Indeed, this underlines the relevance of determining the random error, as investigator-related scattering would also contribute to this kind of error. Additionally, Bates, Dufek & Davis [34] and Dufek, Bates & Davis [35] stressed the role of the sample size for reliability values as well as a lack of generalizability when a testing procedure (in contrast to the reliability analysis of a device) is evaluated. Therefore, the results of this study are not transferable to other studies.

However, the results presented in this study underline the importance of not focusing solely on systematic and relative measurement errors, but rather adopting a more careful and balanced repeatability analysis for different measurements to realistically interpret the study results.

Reliability includes a broad range of indices, including intra- and inter-day repeatability (same conditions, same investigators, different time point), reproducibility (same conditions for the procedure, but different laboratories, investigators etc.), inter-investigator reliability/objectivity (almost the same time point, but different assessors or investigators). Also, validity analyses mostly use the same statistical approaches, comparing values from different measurement systems (e.g., ultrasound vs. magnetic resonance imaging being the gold standard). Given all of these different criteria, an uncertainty regarding the real muscle thickness arises that depends on the magnitude of the calculated value. In this study, the exclusive focus was placed on repeatability, which is just one potential error source, neglecting all other sources. Another origin of variance which might be expressed as secondary variance can be determined between different raters/investigators. A combined investigation approach with multiple test sessions for which data are collected from at least two different investigators was provided by Carstensen [36] and Carstensen et al. [27]. This approach should be applied to follow-up studies to account for further measurement error explorations and with that lead to improvements for future standardization of ultrasound investigations. Unfortunately, these more complex approaches were not suitable in this study, as our data were generated by just one investigator. Additionally, while the 95% confidence bands for the LoA are preferably derived via the exact method [37], we used the approximate approach for a better comparison across scenarios.

Another limitation in this paper stems from the use of horizontal LoAs in the Bland-Altman plot. Heteroscedasticity of data implies that deviations increase when measurement values increase which must be assumed for most sports science and medicine-related parameters [23] and can also be seen in this data collection (see the proportional bias line in Fig. 3). Thus, ideally, the LoAs should adapt to this trend shift and not be completely horizontal (see [38]). However, since this is commonly not done in current sports science and medicine research, this paper also used the simplified, horizontal LoAs. This was inter alia done to improve the comparability to other studies’ results as the focus of this study was to illustrate the shortcomings of current quantifiable parameters in reliability reporting.

The limitations mentioned above should be understood as an outlook and call for future original research to incorporate the latest statistical methods to improve reliability reporting.

## Conclusions

Researchers and clinicians should pay closer attention to random errors when using and referring to pre-post measurement changes using ultrasound-based data collection. The interpretations and derived recommendations should consider the random and systematic measurement error to provide a more careful and reliable statement. Even after accounting for the repeatability measurement source, there is no common classification that relates the different sources of the error to the expected or measured pre-post change, e.g. the magnitude of downscaling for the reported effect sizes or classification of the uncertainty arising from these error sources.

## Data Availability

Data can be provided by the corresponding author on reasonable request.

## Abbreviations

CV: Coefficient of variation
CV_TE_: Coefficient of variation of the typical error
ICC: Intraclass correlation coefficient
GL: Lateral head of the gastrocnemius
GM: Medial head of the gastrocnemius
LoA: Limits of Agreement
M: Mean
MAE: Mean absolute error
MAPE: Mean absolute percentage error
MDC: Minimal detectable change
SD: Standard deviation
SEM: Standard error of measurement
VL: Vastus lateralis
TE: Typical error
